# A retrospective report on xenotransfusion of canine serum albumin in five critically ill cats

**DOI:** 10.3389/fvets.2025.1585988

**Published:** 2025-06-05

**Authors:** Eric Sirabian, Rebecca A. Walton, April E. Blong

**Affiliations:** ^1^College of Veterinary Medicine, Iowa State University, Ames, IA, United States; ^2^VCA West Los Angeles Animal Hospital, Los Angeles, CA, United States

**Keywords:** xenotransfusion, feline, albumin, hypoalbuminemia, canine albumin

## Abstract

This retrospective case series describes the xenotransfusion of canine serum albumin (CSA) in five critically ill cats. Four cats received a single xenotransfusion of CSA, while one cat received two separate xenotransfusions. Overall, two out of five cats (40%) survived long enough to be discharged, which is similar to previously published survival rates for critically ill cats. One cat exhibited clinical signs attributable to acute transfusion-associated circulatory overload (TACO), while two cats showed clinical signs suggestive of a possible, although less likely, transfusion reaction. While a prospective study is needed, the review of the cases presented here suggests that CSA may provide oncotic and hemodynamic support and be safe to administer to critically ill cats.

## Introduction

Albumin is an essential protein with a wide range of functions, such as maintaining oncotic pressure, transporting hormones and drugs, buffering extracellular fluid, regulating microvascular permeability, and preventing thrombus formation (1). Hypoalbuminemia is common and often occurs as a result of critical illnesses, such as sepsis and systemic inflammatory response syndrome (2). Complications associated with hypoalbuminemia include delayed wound healing, an increased risk of gastrointestinal dehiscence, heightened platelet aggregability, development of acute respiratory distress syndrome (2–6), and worsened intravascular volume depletion (2). Fong et al. found that hospitalized cats with moderate or severe hypoalbuminemia (albumin < 23.9 g/L) had a longer duration of hospitalization, higher hospitalization costs, and increased mortality rates compared to those with mild hypoalbuminemia (24–27.9 g/L) (7).

Given the complications associated with hypoalbuminemia, albumin supplementation may be an important focus of patient management during critical illness (2). Hypoalbuminemia may be managed through the administration of species-specific plasma. However, large volumes of plasma are required to provide significant albumin support (8). Alternative options for albumin supplementation include concentrated albumin products. While human serum albumin (HSA) and lyophilized canine serum albumin (CSA) products are available, there are currently no feline-specific albumin products.

Albumin exhibits 83–88% structural homology across various animal species (9). HSA xenotransfusions have been administered to cats with a wide variety of underlying etiologies, with few apparent complications reported in a single prospective study (10). A large-scale retrospective study on HSA administration in 170 cats reported a survival rate of 72%, with symptoms such as diarrhea, hyperthermia, and tremors observed in 33.9% of patients; however, it was not possible to determine which of these symptoms were related to the albumin administration versus the underlying disease process (11). The genetic sequence of CSA is more similar to that of cats than HSA (12). Therefore, CSA may also be a reasonable option for addressing hypoalbuminemia in cats. The administration of CSA to dogs with septic peritonitis resulted in a significant increase in systolic blood pressure, and higher albumin concentrations and was associated with survival to discharge (13). There are currently no documented cases of CSA administration in cats. The purpose of this case series is to describe the xenotransfusion and potential complications associated with the administration of CSA in cats.

## Materials and methods

Electronic records from a single Midwest veterinary teaching hospital were searched between 2016 and 2022. Cases were identified using the charge code for CSA and were then sorted by species. All cats that received CSA at least once were included; there were no exclusion criteria.

The records were reviewed to obtain information on signalment, weight, underlying condition(s), and vital signs before, during, and after xenotransfusion, as well as relevant clinicopathologic parameters—particularly albumin and total plasma protein. General fluid management and other administered transfusion products were also recorded. The final disposition of the patient was also noted. The amount (g/kg) of CSA administered, the concentration at which it was administered, and the duration of administration were recorded when available. Transfusion monitoring records and ongoing medical records were reviewed for any documented evidence of a potential adverse reaction to CSA.

Due to the small number of cases identified, statistical analysis was not possible; therefore, the authors chose to describe the five cases and any potential associated complications as follows.

## Results

A total of five cats aged between 3 months and 17 years were identified as having received at least one CSA transfusion. In four cases, a single CSA xenotransfusion was administered, while the final case received two separate xenotransfusions of CSA. All cats were deemed critically ill based on the presence of hypotension and/or the administration of multiple transfusion products. Overall, two out of five cats (40%) survived to discharge. The relevant case summaries are provided below:

### Case 1

A 17-year-old spayed female domestic shorthair (DSH) cat, weighing 2.5 kg, was presented with a 1-week history of lethargy and anorexia. Upon presentation, the cat was laterally recumbent, hypothermic (36.3°C), tachycardic (250 beats per min), and 8–10% dehydrated. The cat had generalized sarcopenia, icterus, and a palpable abdominal fluid wave. A serum biochemistry profile showed low-normal albumin (ALB; 23 g/L, reference range: 23–39 g/L), hypoproteinemia (total plasma protein; 58 g/L, reference range: 60–80 g/L), hyperbilirubinemia (TBIL; 58 g/L, reference range: 0–9 g/L), and elevated levels of alanine transaminase (ALT; 236 U/L, reference range: 12–130 U/L), alkaline phosphatase (ALKP; 151 U/L, reference range: 14–111 U/L), and gamma-glutamyl transferase (GGT; 13 U/L, reference range: 0–4 U/L). A complete blood count revealed non-regenerative anemia (HCT; 23.9%, reference range: 30–45%) and mature neutrophilia (26.7 K/mcL, reference range: 2.5–12.5 K/mcL). Point-of-care ultrasonography (POCUS) revealed an abdominal fluid score of 4/4. A diagnostic abdominocentesis was performed, and cytology of the abdominal effusion (results reported later in the day) raised suspicion for neoplastic cells. The cat was stabilized with active warming and intravenous boluses of hypertonic saline^1^ (3 ml/kg) and a balanced isotonic crystalloid^2^ (20 ml/kg total). The cat’s blood type was determined (type A) prior to the administration of a type-specific packed red blood cell (pRBC) transfusion (14 ml/kg over 2 h).

A few hours following initial stabilization, the cat became hypotensive, with a systolic blood pressure of 65 mmHg. The cat received a type-A fresh frozen plasma (FFP) transfusion (14 ml/kg over 1 h) and 6 ml/kg/h of balanced crystalloids^2^ for 7 h. The cat remained hypotensive (with a systolic blood pressure of 60 mmHg). Due to progressive abdominal effusion and suspected hypovolemia observed on POCUS, a CSA^3^ transfusion was performed (2 g/kg of 10% CSA over a total of 3 h). However, the blood pressure did not improve and remained at 55 mmHg (systolic). Near the end of the CSA transfusion, the cat was started on norepinephrine^4^ (0.25 mcg/kg/min), and the systolic blood pressure increased to 80 mmHg. Shortly after the administration of CSA, the cat was humanely euthanized due to its deteriorating condition and overall poor prognosis. As a result, there was no opportunity to recheck total plasma protein (TPP) or albumin concentrations. No immediate clinical signs of a transfusion reaction were observed prior to euthanasia.

### Case 2

A 12-year-old male castrated DSH, weighing 4.89 kg, was presented with septic peritonitis based on radiographic evidence of pneumoperitoneum. Upon presentation, the cat was obtunded, tachycardic (280 bpm), and estimated to be 6% dehydrated. The bloodwork revealed anemia with a packed cell volume (PCV) of 28% (reference range: 35–45%), with normal TPP of 74 g/L (reference range: 60–79 g/L) and serum albumin of 24 g/L. The cat was given intravenous fluid boluses of a balanced isotonic crystalloid^2^ (20 ml/kg total), which improved the heart rate to 225 bpm. The systolic blood pressure was 82 mmHg. The cat’s blood type was type A. Based on the cat’s fluid responsiveness, the cat was administered 8.4 ml/kg of type A FFP over 2 h prior to general anesthesia.

An exploratory laparotomy was performed, revealing a jejunal perforation that necessitated a jejunal resection and anastomosis. Histopathology of the tissue was submitted, which later revealed intestinal lymphoblastic lymphoma.

Intraoperative hypotension (mean arterial pressure [MAP]; 48 mmHg) was treated with norepinephrine^4^ (0.1–0.5 mcg/kg/min), dopamine^5^ (5 mcg/kg/min), and a type-specific pRBC transfusion (4 ml/kg over 1 h). Post-transfusion PCV/TPP was 24% and 62 g/L. Postoperatively, the cat was administered 5% CSA^3^ (1 g/kg over 2 h) due to persistent tachycardia (256 bpm) and ongoing hypovolemia assessed by POCUS. After the administration of CSA, tachycardia (228 bpm) and hypotension (systolic 108 mmHg) resolved. One hour after the CSA transfusion, PCV/TPP was 17% and 68 g/L. Albumin remained static at 25 g/L. The cat remained systemically stable and was weaned off vasopressor support. There were no adverse signs noted during or immediately after the CSA transfusion.

The cat remained eupneic until 12 h post-CSA transfusion, at which point it was observed making fish-mouth motions while breathing (assessed as respiratory distress), and pulse oximetry indicated hypoxemia (89–90%) on room air. Thoracic radiographs, interpreted by a radiologist, revealed collapse of the right cranial and middle lung lobes with associated mediastinal shift and no radiographic evidence of pulmonary vein distention or fluid overload. The remaining lung fields appeared normal. The radiologist provided differential diagnosis, included pneumonia and pulmonary thromboembolism. The cat was already on antibiotics to address the septic peritonitis, so enoxaparin^6^ (0.8 mg/kg SQ q8h) was started to address possible thromboembolism. The cat remained on intravenous fluids/drugs, totaling 35 ml/kg over 24 h, and did not receive diuretic therapy. Radiographs taken the day following the onset of respiratory symptoms indicated complete resolution of the previously collapsed lung lobes. An echocardiogram performed on the same day revealed mild right atrial, left atrial, and left ventricular enlargement, which was suspected to be secondary to fluid administration. Supplemental oxygen was discontinued without the addition of other treatments, and the cat was discharged 5 days after presentation.

### Case 3

A two-year-old neutered male DSH cat, weighing 3.3 kg, was presented for postoperative management following an exploratory laparotomy. One day prior to presentation, an abdominal laparotomy was performed by the primary veterinarian. Biopsies of the pancreas, stomach, and jejunum were obtained, which later confirmed lymphoplasmacytic and eosinophilic enteritis. The cat was discharged but returned the next day due to bleeding from the incision and extensive bruising. The cat was placed under general anesthesia, and a second exploratory laparotomy was performed, which revealed subcutaneous hemorrhage, as well as hemorrhage from the pancreatic biopsy site and omentum. There was dehiscence at one of the suture sites from the intestinal biopsy that was closed. The cat was given fresh plasma and vitamin K (unknown doses) and was then transferred to the University for further diagnostic testing and therapy.

Upon presentation, the cat was quiet, hypothermic (35.4°C), tachypneic (50 breaths per min), and had a heart rate of 200 bpm. It exhibited pale mucous membranes, delayed capillary refill time, and weak pulses. Pain was noted on abdominal palpation. Isotonic crystalloid fluid^2^ (30 ml/kg total) and hypertonic saline^1^ (4.5 ml/kg) were administered as intravenous bolus therapy. After 6 h, progressive abdominal effusion was noted on POCUS. Cytology from an abdominocentesis sample revealed septic suppurative inflammation. The bloodwork revealed a normal leukocyte count with a left shift (immature neutrophils 0.553 K/mcL, reference range: 0–0.3 K/mcL), hypoalbuminemia (17 g/L), and hypoproteinemia (35 g/L). The cat’s blood type was type A.

The cat was anesthetized for a third exploratory laparotomy. The cat became hypotensive (MAP of 45 mmHg) during general anesthesia; therefore, norepinephrine^4^ (0.5–1.5 mcg/kg/min) was started. A type A FFP transfusion (10 ml/kg over 5 min) was administered, followed by a second FFP transfusion (9 ml/kg over 1 h) to treat persistent hypotension. A type-specific pRBC transfusion was also started intraoperatively (9 ml/kg over 3 h). The previous pancreatic biopsy site had a mature hematoma with a small amount of purulent discharge.

Immediately postoperatively, the cat was hyperlactatemic (4.68 mmol/L, reference range: 0–2.5 mmol/L). The mean arterial blood pressure was 66 mmHg. A third FFP transfusion was administered (18 ml/kg over 4 h) along with CSA^3^ (1.5 g/kg in a 10% solution over 1 h). No transfusion reactions were observed. The blood pressure increased (MAP 85 mmHg) during the CSA transfusion. Total plasma protein increased to 54 g/L 6 h after completing the CSA transfusion, while serum albumin increased to 28 g/L 24 h after the transfusion.

The following day, hypotension recurred (60 mmHg systolic), and an additional transfusion of FFP (8 ml/kg over 3 h) was administered along with norepinephrine (0.25 mcg/kg/min). The systolic blood pressure improved to 102 mmHg.

On day 4 of hospitalization, 60 h after the initial CSA^3^ transfusion, the cat received a second CSA transfusion (1.5 g/kg in a 7% solution over 4 h) due to recurrent hypotension (70 mmHg systolic). The systolic blood pressure increased to 80 mmHg after the CSA transfusion. Total plasma protein was 42 g/L prior to the transfusion and 57 g/L when measured 3 h after completing the CSA transfusion. The serum albumin pre-transfusion was 17 g/L and improved to 27 g/L 18 h after completing the CSA transfusion. No immediate concerns regarding a transfusion reaction were noted.

Four hours after the second CSA transfusion, the cat was tachypneic and hypoxemic (SpO2 90% on room air). Thoracic radiographs revealed pulmonary edema and pulmonary vessel enlargement. The cat was treated with supplemental oxygen (FiO_2_ 40%). Due to suspected transfusion-associated circulatory overload (TACO), furosemide^7^ was administered as a continuous intravenous infusion (2.5 mg/kg cumulatively over 6 h), and the cat was given pimobendan^8^ (0.19 mg/kg orally every 12 h). The following day, an echocardiogram demonstrated no signs of intracardiac volume overload, and the cat showed clinical improvement. The cat was gradually weaned off intensive care and monitoring prior to discharge on day 10 of hospitalization.

### Case 4

A 2-year-old male castrated DSH cat, weighing 6.8 kg, was presented for continued management of a possible uroabdomen. Five days before the presentation, the cat was diagnosed with a urethral obstruction. The primary veterinarian placed a urinary catheter and hospitalized the cat with IV fluids, analgesia, and antibiotics. The urinary catheter became non-patent, prompting referral.

On presentation, the cat was tachypneic (60 breaths per min) and dyspneic. The rectal temperature was elevated at 40.1°C. The bloodwork revealed hypoproteinemia (ALB 15 g/L and TPP 52 g/L) and anemia (PCV 19%). The cat had moderate-to-severe peripheral edema on its ventral abdomen and perineum, as well as pleural and peritoneal effusion identified on POCUS. Abdominocentesis and cytology confirmed non-septic fluid with mild neutrophilic inflammation. An attempt at retrograde urethral catheterization was unsuccessful. A positive contrast retrograde urethrocystogram confirmed a urethral tear at the level of the pelvic inlet.

A therapeutic thoracocentesis was performed, which improved the respiratory rate from 60 to 40 breaths per min. The cat’s blood type was type A. Perioperatively, the cat was given a type A FFP transfusion (4.4 ml/kg over 2 h), as well as a CSA^3^ transfusion (0.7 g/kg, concentration not noted) over 4 h for oncotic support. The cat was anesthetized for an exploratory laparotomy to place a normograde urinary catheter. The cat was on an anesthetic ventilator during the procedure and required norepinephrine (0.1–0.5 mcg/kg/min) to maintain normotension. Postoperatively, the cat’s PCV was 17%. The cat received 10 ml/kg of type A fresh whole blood (FWB) to address its anemia. The cat’s TPP postoperatively and post-CSA was 60 g/L. Postoperatively, the cat was also normotensive (MAP 82) without norepinephrine. Furthermore, 2 h after discontinuing anesthesia and 3 h after receiving CSA, the cat remained unresponsive and hypercapneic (P_a_CO_2_ 73 mmHg) without ventilatory support. The cat’s P_a_O_2_: F_i_O_2_ ratio was 198 (F_i_O_2_ 1.0). It had crackles on auscultation of the right middle and left ventral hemithorax. Due to the cat’s lack of recovery from anesthesia and the requirement for mechanical ventilation as a result of hypoventilation, the owner opted to withdraw care, allowing the cat to pass on its own under heavy sedation.

### Case 5

A 3-month-old female DSH cat, weighing 0.82 kg, was presented in septic shock secondary to severe ulcerative pyoderma caused by a multidrug-resistant *Staphylococcus pseudintermedius*. Prior to receiving culture results, the cat was treated with oral amoxicillin/clavulanate potassium^9^ (15.6 mg/kg twice daily) and methyprednisolone^10^ (2 mg/kg/day × 3 days) to address a possible immune-mediated etiology of her disease.

On presentation, the cat was laterally recumbent, stuporous, bradycardic (heart rate at 110 bpm), and hypothermic (33.3°C). Both blood glucose and blood pressure were too low to read. Initial treatments for stabilization included warming, a balanced isotonic crystalloid^2^ bolus (30 ml/kg), a 50% dextrose^11^ bolus (1 ml/kg), and intravenous atropine^12^ (0.048 mg/kg).

The cat was hospitalized and started on supportive therapy, in addition to parenteral sulfadiazine/trimethoprim^13^ (14 mg/kg IV every 12 h), based on previous culture and susceptibility results. Over 24 h of hospitalization, the albumin level decreased from 28 g/L to 15 g/L. The PCV/TPP level was 26% and 60 g/L. The cat became hypotensive (48 mmHg systolic). Blood type testing was performed, and the cat was determined to be blood type A. Norepinephrine^4^ (0.25 mcg/kg/min) was started, but there was no apparent improvement. A type-specific FFP transfusion was administered (20 ml/kg over 2 h) due to a lack of response to norepinephrine and suspected hypovolemia from skin exudate. The kitten remained hypotensive, so CSA^3^ was administered (1.4 g/kg of a 10% solution) over 3 h to augment effective circulating volume. The cat remained hypotensive (55 mmHg systolic blood pressure); however, no immediate transfusion reactions were noted. Two hours after the beginning of the CSA transfusion, and prior to its completion, humane euthanasia was selected due to the overall poor condition and guarded prognosis. Due to the timing of euthanasia, no post-transfusion TPP or albumin values were available.

## Discussion

This retrospective case series describes the xenotransfusion of CSA to five cats. Six separate transfusions were given to the five cats due to hemodynamic instability and the need to provide oncotic support. A total of three of the five cats demonstrated a positive short-term response to lyophilized CSA transfusion. A total of three of the six (50%) CSA transfusions led to improved blood pressure in hypotensive cats (Cases 2 and 3), while two did not result in any change (Cases 1 and 5), and one animal remained normotensive once anesthesia was discontinued (Case 4). In addition, three of the six (50%) CSA transfusions resulted in documented increases in TPP and/or albumin concentrations. In Case 2, the TPP level was the same at intake and postoperatively, after albumin was administered. The authors suspect this value remained unchanged due to fluid resuscitation and a balance between albumin administration and further perioperative loss. The final two cats did not have post-CSA TPP or albumin concentrations available due to their prompt euthanasia during or after CSA administration. The overall survival rate in the population of the cats that received CSA was low, at only 40% (two of the five cats). However, all five cats experienced at least transient hypotension that required vasopressor therapy. Other studies evaluating outcomes in critically ill cats with hypotension have reported survival rates ranging between 15 and 39% (14, 15). Therefore, it should not be concluded that the low survival rate reported was directly related to CSA administration, rather than the overall level of illness present in this population of cats.

In accordance with the recent Transfusion Reaction Small Animal Consensus Statement (TRACS), there are four types of transfusion reactions that can result in respiratory compromise (16). Allergic transfusion reactions are type-I immunologic reactions that occur during or within 4 h of transfusion, resulting in anaphylaxis symptoms. In cats, this commonly presents as respiratory distress but can also manifest as gastrointestinal upset or dermatologic signs. TACO is a non-immunologic reaction that develops within 6 h of transfusion due to increased blood volume. Evidence of increased hydrostatic pressure and pulmonary edema is needed, and a positive response to diuretic therapy is expected. Transfusion-related acute lung injury (TRALI) is an acute (within 6 h), immunologically mediated reaction resulting in hypoxemia and non-cardiogenic pulmonary edema. Transfusion-associated dyspnea (TAD) is an acute transfusion reaction that results in respiratory distress within 24 h of transfusion and requires the exclusion of TACO, TRALI, allergic reactions, and other pulmonary pathologies (16). In addition to defining transfusion reactions, TRACS also provides guidelines for determining the imputability of these reactions. The guidelines classify reactions as definite, meaning there are no other causes of the symptoms; probable, meaning there are other possible causes but transfusion is the most likely; and possible, meaning other causes are more likely but transfusion cannot be ruled out (16).

In the current case series, there were three possible CSA transfusion reactions, primarily exhibited by respiratory signs (i.e., hypoxemia, tachypnea, or hypercapnia). The onset of symptoms began 3–12 h after the administration of CSA. Possible transfusion-related differentials in these cases of respiratory distress included acute allergic reaction, TACO, TRALI, and TAD. Other non-transfusion-related differentials in these cases included pneumonia, pulmonary thromboembolism, and acute respiratory distress syndrome. The authors applied the TRACS criteria and imputability to each case.

Case 3 developed respiratory signs within 4 h of CSA transfusion and had documented pulmonary edema and pulmonary venous distention on radiographs. The cat also improved after diuretic therapy, indicating definite TACO as per the TRACS guidelines. Case 2 developed respiratory distress 12 h after CSA transfusion and exhibited focal unilateral radiographic changes. The condition resolved rapidly (within 24 h) with no specific treatment, other than the initiation of thromboprophylaxis. Given the timeline, TAD is the only potential transfusion-related cause of respiratory symptoms that could be applicable. Radiographs revealed clear pulmonary pathology, suggesting possible TAD. While TAD cannot be ruled out, cases of TAD would not be expected to demonstrate focal radiographic pulmonary abnormalities (17). Finally, in Case 4, the cause of progressive respiratory dysfunction was not clear due to the cat being euthanized. However, given that the cat failed to regain consciousness following the cessation of anesthesia and reversal of all drugs and remained hypoventilatory, a central neurologic cause of its failed recovery is suspected. However, given the auscultated pulmonary crackles, a component of TACO, TRALI, or another non-transfusion-related cause of its decreased lung function may have also been present. As no imaging was available for this cat, it is not possible to determine whether TACO or TRALI was more likely, but either is possible. This cat received other blood component therapy at the same time as CSA, which makes it impossible to determine whether the CSA or other blood products could have resulted in TACO or TRALI, if present.

Given the retrospective nature of this report, there are many limitations to this study. All cats were deemed critically ill, and there was no opportunity for post-CSA assessment of protein concentration in two of the five cases. In addition, no long-term follow-up was available for any case, precluding the identification of delayed transfusion reactions. The retrospective nature of the report also makes it difficult to determine whether any changes in symptoms were solely due to CSA, other blood products, or progression of the underlying disease. With the exception of suspected TACO in Case 3, the authors suspect that the remaining symptoms reported in this case series were likely the result of the underlying disease process rather than the CSA directly. Future directions include prospective studies to validate the benefits of CSA administration in cats and more completely document any transfusion reactions. In this case series, we conclude that lyophilized CSA is likely a reasonable option for feline patients who may benefit from oncotic or hemodynamic support, although it is not without risks, similar to other transfusion products.

## Data Availability

These are medical records which are confidential. Requests to access these datasets should be directed to aeb287@iastate.edu.

## Footnotes

^1^Hypertonic saline 7.2%, VetOne, MWI, Boise ID.

^2^Normosol-R, ICU Medical Inc., Lake Forrest, IL.

^3^Canine albumin, lyophilized, Animal Blood Resources International, Dixon, CA.

^4^Norepinephrine bitartrate, Baxter Healthcare, Duluth, GA.

^5^Dopamine HCl, Hospira Inc., Lake Forrest, IL.

^6^Enoxaparin, Northstar Rx LLC, Memphis, TN.

^7^Salix, Merck Animal Health, Rahway, NJ.

^8^Vetmedin, Boehringer Ingelheim, Duluth, GA.

^9^Clavamox drops, Zoetis, Kalamazoo, MI.

^10^Medrol, Zoetis, Kalamazoo, MI.

^11^Dextrose 50%, VetOne, MWI, Boise, ID.

^12^Atropine sulfate, Fresenius Kabi, Lake Zurich, IL.

^13^Sulfamethoxazole and trimethoprim injection, Teva Pharmaceuticals, Sellersville, PA.
